# ATP-Induced Contractile Response of Esophageal Smooth Muscle in Mice

**DOI:** 10.3390/ijms25041985

**Published:** 2024-02-06

**Authors:** Yuji Suzuki, Yasutake Shimizu, Takahiko Shiina

**Affiliations:** 1 Department of Basic Veterinary Science, Laboratory of Physiology, Joint Graduate School of Veterinary Sciences, Gifu University, 1-1 Yanagido, Gifu 501-1193, Japanshimizu.yasutake.b5@f.gifu-u.ac.jp (Y.S.); 2 Division of Animal Medical Science, Center for One Medicine Innovative Translational Research (COMIT), Gifu University Institute for Advanced Study, 1-1 Yanagido, Gifu 501-1193, Japan

**Keywords:** ATP, esophagus, motility, mouse, smooth muscle

## Abstract

The tunica muscularis of mammalian esophagi is composed of striated muscle and smooth muscle. Contraction of the esophageal striated muscle portion is mainly controlled by cholinergic neurons. On the other hand, smooth muscle contraction and relaxation are controlled not only by cholinergic components but also by non-cholinergic components in the esophagus. Adenosine triphosphate (ATP) is known to regulate smooth muscle contraction and relaxation in the gastrointestinal tract via purinergic receptors. However, the precise mechanism of purinergic regulation in the esophagus is still unclear. Therefore, the aim of the present study was to clarify the effects of ATP on the mechanical responses of the esophageal muscle in mice. An isolated segment of the mouse esophagus was placed in a Magnus’s tube and longitudinal mechanical responses were recorded. Exogenous application of ATP induced contractile responses in the esophageal preparations. Tetrodotoxin, a blocker of voltage-dependent sodium channels in neurons and striated muscle, did not affect the ATP-induced contraction. The ATP-evoked contraction was blocked by pretreatment with suramin, a purinergic receptor antagonist. RT-PCR revealed the expression of mRNA of purinergic receptor genes in the mouse esophageal tissue. The findings suggest that purinergic signaling might regulate the motor activity of mouse esophageal smooth muscle.

## 1. Introduction

Motor functions of the gastrointestinal tract are regulated by various transmitters including purines such as adenosine, adenosine diphosphate (ADP) and adenosine triphosphate (ATP) [1,2,3,4,5]. There are two types of purine receptors: one type are P1 receptors for adenosine and the other type are P2 receptors for ATP and ADP [1,2,6,7]. P2 receptors include P2X receptors and P2Y receptors. P2X receptors are ligand-gated ion channels and P2Y receptors are G protein-coupled receptors (GPCRs) [1,2,6,7]. At present, seven P2X (P2X1-7) and eight P2Y (P2Y1, 2, 4, 6, and 11–14) receptor subtypes have been identified [8]. However, there are also some reports that rodents such as rats and mice do not have P2Y11 receptors [9]. P2 receptors are expressed in the gastrointestinal tract, including the esophagus [2,8,10,11,12,13]. Previous studies have demonstrated that purines cause excitatory and inhibitory responses in the smooth muscle cells of the gastrointestinal tract [2,8,11,14,15]. 

The tunica muscularis of mammalian esophagi contains not only smooth muscle fibers but also striated muscle fibers [16,17]. For example, the upper portion is composed of striated muscle fibers, the lower portion is composed of smooth muscle fibers, and the middle portion is a mixed composition of these fibers in human and cat esophagi [17]. On the other hand, in dogs, ruminants, and rodents, including mice, rats and hamsters, the muscle layer of the esophagus consists largely of striated muscle fibers [17]. The motility of the esophagus is controlled by extrinsic cholinergic vagal neurons [18,19] and by intrinsic myenteric neurons releasing various neurotransmitters such as acetylcholine, tachykinins, nitric oxide, and galanin [17,19,20,21,22]. In the case of striated muscles, cholinergic receptors are nicotinic receptors, while in the case of smooth muscles, they are muscarinic receptors. In addition, the esophagus has longitudinal smooth muscle fibers in the muscularis mucosae even in mammals that have a striated muscle esophagus [23,24]. The mouse esophagus also contains a smooth muscle layer in the muscularis mucosae. The esophageal muscularis mucosa is used for the study of smooth muscle contractility in the esophagus [23,24,25,26,27].

Purinergic receptors have been detected histologically in the esophagus [11,12]. Although there have been functional studies on the purinergic regulation of esophageal smooth muscle [27,28], the precise mechanism is still unclear. Therefore, the aim of the present study was to determine the characteristics of mechanical responses induced by ATP in the mouse esophagus.

## 2. Results

### 2.1. Molecular Identification of P2 Receptors in the Mouse Esophagus

**Figure 1:**
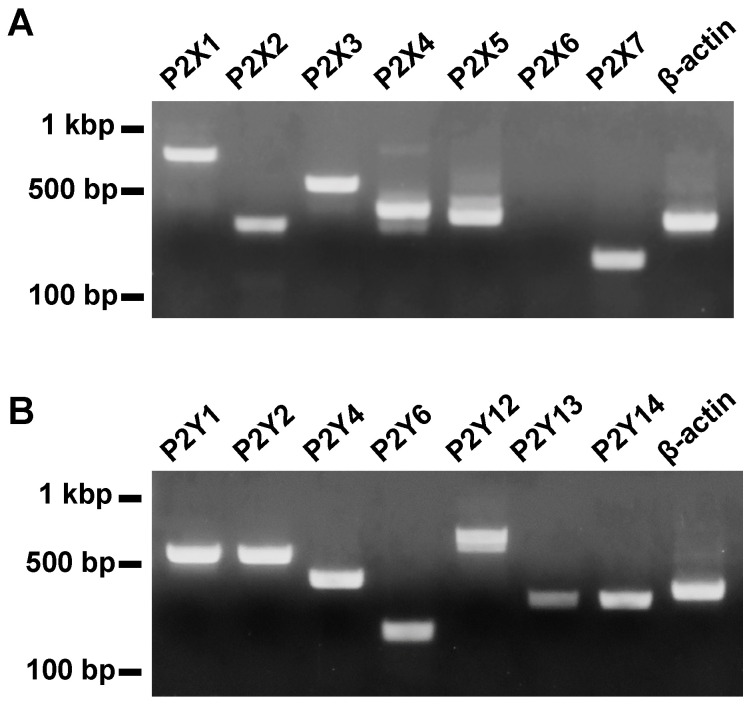
Expression of subtypes of P2 receptors in the mouse esophageal tissue determined by RT-PCR. Amplified products of mRNA of P2X1, P2X2, P2X3, P2X4, P2X5, P2X6, P2X7, P2Y1, P2Y2, P2Y4, P2Y6, P2Y12, P2Y13, P2Y14, and β-actin were detected in appropriate sizes (*n* = 3). (**A**) Shows expression of P2X receptors and β-actin. (**B**) Shows expression of P2Y receptors and β-actin.

We examined the expression of subtypes of P2 receptors in mouse esophageal tissues by using reverse transcription polymerase chain reaction (RT-PCR). Amplified products of mRNA of P2X1, 2, 3, 4, 5, and 7 receptors and P2Y1, 2, 4, 6, 12, 13, and 14 receptors were observed in appropriate sizes (Figure 1).

### 2.2. Effects of ATP on the Mechanical Activity of Mouse Esophageal Segments

**Figure 2:**
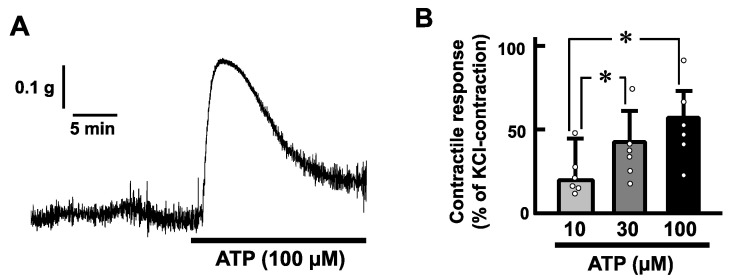
ATP-evoked contractions in the mouse esophagus. (**A**) A representative trace demonstrating the effect of ATP on longitudinal tension of the mouse esophagus is shown. (**B**) Dose-dependency of the contractile responses evoked by ATP in the mouse esophagus is summarized (*n* = 6). The values of contractile responses are normalized as percentages of KCl (60 mM)-induced contractions. Each bar represents the mean of data ± standard error of the mean (S.E.M.). * *p* < 0.05.

**Figure 3:**
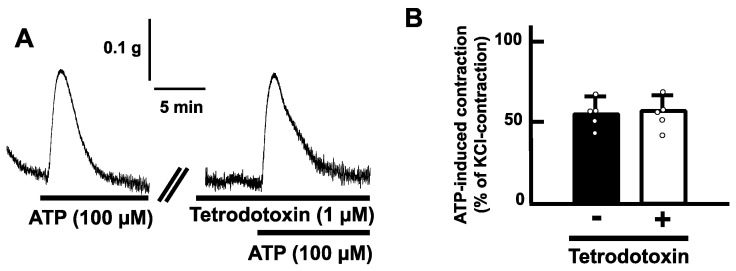
Effects of tetrodotoxin on ATP-evoked contraction in the mouse esophagus. (**A**) Representative traces of the contraction induced by ATP (100 µM) of the mouse esophagus in the absence or presence of tetrodotoxin (1 µM) are shown. (**B**) Summarized bar graphs of contraction evoked by ATP (100 µM) of the mouse esophagus in the absence (−) or presence (+) of tetrodotoxin (1 µM) are shown (*n* = 5). The values of contractile responses are normalized as percentages of KCl (60 mM)-induced contractions. Each bar graph represents the mean of data ± S.E.M.

Exogenous application of ATP (100 µM) induced a transient contraction in a longitudinal direction of the mouse esophageal segments (Figure 2A). The contractile responses increased in a concentration-dependent manner (Figure 2B). On the other hand, tetrodotoxin (1 µM) did not affect the ATP-induced contractions of the mouse esophagus (Figure 3).

### 2.3. Effects of Purinoceptor Antagonists on ATP-Evoked Contractions in the Mouse Esophagus

**Figure 4:**
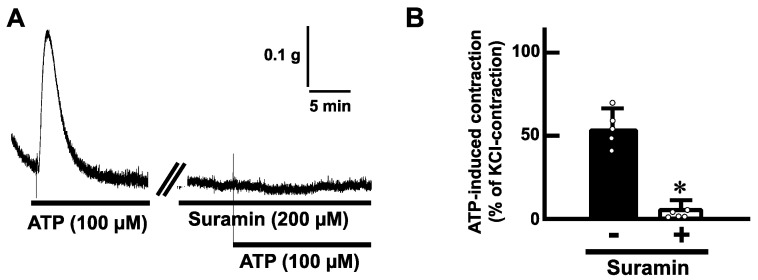
Effects of a non-selective antagonist for P2 receptors on ATP-evoked contraction in the mouse esophagus. (**A**) Representative tracings demonstrating the contraction induced by ATP (100 µM) in the mouse esophagus under the absence or presence of suramin (200 µM) are shown. (**B**) Summarized bar graphs of contraction evoked by ATP (100 µM) of the mouse esophagus in the absence (−) or presence (+) of suramin (200 µM) are shown (*n* = 5). The values of contractile responses are normalized as percentages of KCl (60 mM)-induced contractions. Each bar represents the mean of data ± S.E.M. * *p* < 0.05, compared to the control.

**Figure 5:**
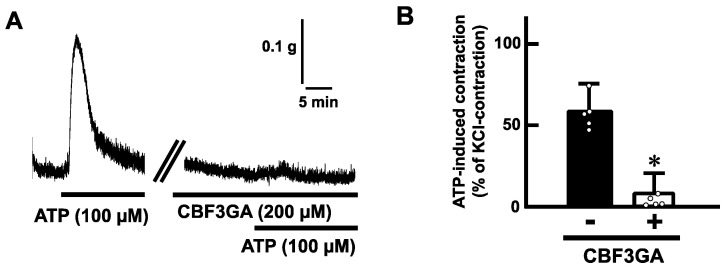
Effects of a selective antagonist for P2Y receptors on ATP-evoked contraction in the mouse esophagus. (**A**) Representative tracings demonstrating the contraction induced by ATP (100 µM) of the mouse esophagus in the absence or presence of CBF3GA (200 µM) are shown. (**B**) Summarized bar graphs of contraction evoked by ATP (100 µM) of the mouse esophagus in the absence (−) or presence (+) of CBF3GA (200 µM) are shown (*n* = 5). The values of contractile responses are normalized as percentages of KCl (60 mM)-induced contractions. Each bar represents the mean of data ± S.E.M. * *p* < 0.05, compared to the control.

To determine whether ATP-evoked contractions are mediated via purinoceptors, we examined the effects of antagonists for purinoceptors. Pretreatment with suramin (200 µM), a non-selective P2 receptor antagonist, blocked the ATP (100 µM)-induced contractions in mouse esophageal segments (Figure 4). Next, we tested a selective antagonist for the P2Y receptor. Pretreatment with cibacron blue F3GA (CBF3GA) (200 µM), a P2Y receptor antagonist, inhibited the ATP-evoked contractions (Figure 5).

## 3. Discussion

In the present study, we investigated the characteristics of mechanical responses induced by ATP in the mouse esophagus. Our major findings are: (1) we detected expression of P2 receptors in the mouse esophagus, (2) exogenous application of ATP evoked contractions of the esophageal smooth muscle, and (3) pretreatment with a selective P2Y receptor antagonist inhibited the ATP-induced contraction. These findings suggest that ATP is involved in excitatory regulation of the longitudinal smooth muscle in the muscularis mucosae of the mouse esophagus via P2Y receptors.

The muscle composition of the tunica muscularis is striated muscle fiber in the mouse esophagus [17]. In addition, the mouse esophagus also contains a smooth muscle layer in the muscularis mucosae. The smooth muscle layer in the muscularis mucosae is longitudinally arranged and thus can express longitudinal mechanical responses exclusively [23,29,30]. Our results showed that application of ATP evoked contraction longitudinally in the esophageal segments. To determine whether ATP acts on smooth muscle or striated muscle, we used tetrodotoxin, which can inhibit contractile activity of striated muscle via blockade of voltage-dependent sodium channels. Pretreatment with tetrodotoxin did not affect the ATP-induced contraction. The concentration of tetrodotoxin used in this study (1 µM) is enough to abolish esophageal striated muscle contractility [23]. Hence, it is reasonable that ATP-induced contraction in the mouse esophagus is a smooth muscle activity in the muscularis mucosae. In addition, since tetrodotoxin can block neuronal activity, the possibility of involvement of neurons in ATP-induced contraction also might be ruled out.

Pretreatment with application of a P2Y receptor antagonist inhibited the ATP-induced contraction in the mouse esophagus. The results indicate that P2Y receptors are involved in ATP-induced contraction of the mouse esophagus. It is known that ATP causes excitatory responses in the smooth muscle cells of other gastrointestinal tracts [2,8,14,15]. The esophagus might have a similar regulation system via P2Y receptors. P2Y receptors are GPCRs [1,2,6,7]. P2Y1, 2, 4, and 6 are coupled preferentially with Gq/_11_ protein [1], which can activate phospholipase C, generate inositol trisphosphate, increase intracellular calcium, or generate diacylglycerol, and activate protein kinase C and then lead to smooth muscle contractions [29]. ATP acts on several P2Y receptors with high affinity [1]. Further investigation is required to identify the subtype of P2Y receptors involved in the excitatory regulation by ATP in esophageal motility.

In previous studies, the mouse esophagus contained P2X3 receptors in the myenteric neurons [12]. If exogenous application of ATP acts on the P2X3 receptors, P2X3 receptor-positive myenteric neurons might be activated, which might induce responses in the esophagus. In this study, however, ATP-induced contractile responses were not affected by pretreatment with tetrodotoxin, which is a blocker of voltage-dependent sodium channels in the neurons. In addition, ATP-induced contractile responses were inhibited by pretreatment with the P2Y receptor antagonist. The results show that our obtained data are not associated with the biological activity of P2X3 receptors in the myenteric neurons. In future, examination of the roles of P2X receptors in the esophagus should be performed to advance studies on purinergic regulation of esophageal motor functions.

In this study, we did not identify endogenous sources of ATP in the mouse esophagus. Interestingly, Mihara et al. reported that ATP is released from epithelial keratinocytes in the mouse esophagus in response to TRPV4 activation [31,32]. We therefore consider that epithelial cells are candidate sources. However, it should be noted that we cannot exclude neurons, glial cells, and muscle cells as sources of ATP. Indeed, it is known that skeletal muscle releases ATP via pannexin channels during repetitive contraction [33].

We have identified various regulatory factors in esophageal motility including tachykinins, nitric oxide, galanin, serotonin [20,22,23,24], and purines in this study. Generally, it is important to control contraction and/or relaxation in the longitudinal direction separately from those in the circular direction for effective peristalsis of the gastrointestinal tract [3,34]. In esophageal peristaltic activity, the longitudinal motor response plays an important role and assists effective propulsion [35]. Our findings suggest that the purinergic system may contribute to effective propulsion in the esophagus: whereas, it should be noted that our experiments were performed using isolated esophageal segments in the organ bath. To advance investigation of purinergic regulation of esophageal motility, an in vivo experimental system is necessary. This is because esophageal motility is caused by the vagus nerve reflex that is integrated in the medulla oblongata [18,19]. We devised an in vivo system for recording esophageal motor activity [36], and this system can be used for further experiments. 

In the human esophagus, the upper portion is composed of striated muscle fibers, the lower portion is composed of smooth muscle fibers, and the middle portion is a mixed composition of these fibers [17]. In mice, on the other hand, the muscle layer of the esophagus consists largely of striated muscle fibers [17]. However, longitudinal smooth muscle fibers in the muscularis mucosae commonly exist in both the human and mouse esophagus [18,19]. So, the findings obtained in this study can be applied to human healthcare. 

In conclusion, the present study clarified that ATP induces contractile responses of longitudinal smooth muscle in the muscularis mucosae of the mouse esophagus via P2Y receptors. This purinergic regulation might contribute to esophageal motility. 

## 4. Materials and Methods

### 4.1. Animals

Male ddY mice (*Mus musculus*, 8–12 weeks of age, 30–40 g in weight) were purchased from Japan SLC (Shizuoka, Japan). The number of animals used was 23. They were maintained in plastic cages at 24 ± 2 °C with a 12:12 h light–dark cycle (light on at 07:00–19:00) and given free access to laboratory chow and water. The experiments were approved by the Gifu University Animal Care and Use Committee and were conducted in accordance with the committee guidelines on animal care and use (permission numbers: H30-183, 2019-239, 2020-252). The issuance and expiry date of the license are 1 April 2019 and 31 March 2022, respectively. 

### 4.2. Esophageal Tissue Preparations

The mice were anesthetized with isoflurane and were exsanguinated via axillary arteries. We isolated a 1 cm long segment from the middle thoracic part of the esophagus. The segment of the esophagus was immediately immersed in Krebs’s solution (see below) at room temperature, and the intraluminal contents of the excised segment were flushed using a small cannula containing Krebs’s solution.

### 4.3. Recording of Mechanical Activity in Esophageal Segments

To record contractile responses in the longitudinal direction, the whole segment was mounted in a Magnus’s tube (10 mL in capacity) filled with Krebs’s solution (pH 7.4). One end of the esophageal segment was tied to the Magnus’s tube and the other end was secured with a silk thread to an isometric force transducer (T7-8-240, Orientec, Tokyo, Japan). The Krebs’s solution was continuously bubbled with a 95% O_2_ + 5% CO_2_ gas mixture and maintained at 37 °C. Mechanical responses, which were filtered and amplified by an amplifier (NEC, AS1202, Tokyo, Japan), were recorded using a PowerLab system (AD Instruments, Bella Vista, NSW, Australia). An initial resting tension of 1.0 g was added to the esophageal segment, which was subsequently allowed to equilibrate for at least 30 min. The esophageal segments were used for experiments for at least 6 h.

### 4.4. Solutions and Drugs

During the experiments, tissues were maintained in Krebs’s solution consisting of (mM): NaCl 118.4, KCl 4.7, CaCl_2_ 2.5, MgSO_4_ 1.2, KH_2_PO_4_ 1.2, NaHCO_3_ 25, and glucose 11.7. ATP was obtained from Tokyo Chemical Industry (Tokyo, Japan). Tetrodotoxin was obtained from FUJIFILM Wako (Osaka, Japan). Suramin and CBF3GA, which were used as antagonists for purinergic receptors [37,38], were obtained from Sigma-Aldrich (St Louis, MO, USA). The drugs were dissolved in distilled water. We confirmed that the highest concentration of vehicles (0.1%) for the drugs alone had no effect on the basal tone and contractile responses at the concentrations used. Final concentrations in the bath solution were described as the concentrations of drugs. 

### 4.5. RNA Isolation and RT-PCR

The expression of P2 receptor gene mRNAs was assessed by RT-PCR. Total cellular RNA was extracted from homogenates of the mouse esophageal mucosa tissue using TRI Reagent (Molecular Research Center, Cincinnati, OH, USA). First-strand cDNA was synthesized from 2 µg of total RNA by using SuperScript III Reverse Transcriptase (Thermo Fisher Scientific, Waltham, MA, USA) and Random primers (Thermo Fisher Scientific). PCR was performed with Platinum Taq DNA Polymerase High Fidelity (Thermo Fisher Scientific). The primer sets are shown in Table 1. All primers were designed according to previous reports [39,40] and obtained from Thermo Fisher Scientific. Amplifications were performed by 35 cycles. The reaction products were electrophoresed on 1.5% agarose gels and stained with ethidium bromide (0.8 µg/mL). The gels were imaged with a UV transilluminator (UVP Laboratory Products, Upland, CA, USA) and photographed.

### 4.6. Data Analysis

Data are presented as means ± standard error of the mean (S.E.M.). *n* indicates the number of separate preparations. The values of contractile responses are maximum amplitudes of contractions induced by application of ATP that are normalized as percentages of KCl (60 mM)-induced contractions in the same preparations. KCl was applied into Krebs’s solution at the latest time point of each experiment. The significance of differences between mean values was determined by one-way analysis of variance followed by the Turkey–Kramer test for the comparison of multiple groups or by the paired *t*-test for comparison of two groups. A *p* value less than 0.05 denotes the presence of a statistically significant difference. 

## Figures and Tables

**Table 1:** List of primers for RT-PCR.

Gene	Sequence (5′-3′)	Predicted Size (bp)
P2X1	Forward CATTGTGCAGAGAACCCAGAA Reverse ATGTCCTCCGCATACTTGAAC	776
P2X2	Forward ACGTTCATGAACAAAAACAAG Reverse TCAAAGTTGGGCCAAACCTTTGG	360
P2X3	Forward AAGAGTGGGCAGTTACAAGGG Reverse GAAAACCCACCCCACAAAGT	576
P2X4	Forward GAGAATGACGCTGGTGTGCC Reverse TTGGTGAGTGTGCGTTGCTC	437
P2X5	Forward TAGTTAATGGCAAGGCGGGA Reverse AGCTCTGGCTACGTCTTCAC	409
P2X6	Forward TACGTACTAACAGACGCA Reverse ATATCAGGGTTCTTTGGG	254
P2X7	Forward TGTTTCCTTTGGCTGCTCCT Reverse CGCTCACCAAAGCAAAGCTAAT	239
P2Y1	Forward TGGCGTGGTGTACCCTCTCAAGTC Reverse CGGGACAGTCTCCTTCTGAATGTA	558
P2Y2	Forward CTCACGCGCACCCTCTACTA Reverse TCGGGTGCACTGCCTTTCTT	549
P2Y4	Forward CTTTGGCTTTCCCTTCTTGA Reverse GTCCGCCCACCTGCTGAT	427
P2Y6	Forward GCCCTGTGCTGGAGACCTTC Reverse CATGGCCCCAGTGACAAACA	226
P2Y12	Forward CAGTGCAAGGGGTGGCATCT Reverse TGGCACACCAAGGTTCTCAG	618
P2Y13	Forward GAAGAGAGGCACATGCAACA Reverse TTACTAATGCCAGGCCAACC	345
P2Y14	Forward CAGTGCATGGAGCTCAAAAA Reverse GCAGCCGAGAGTAGCAGAGT	347
β-actin	Forward TGACCCTGAAGTACCCCATTG Reverse TCAGGATCTTCATGAGGTAG	387

## Data Availability

The data presented in this study are available on request from the corresponding author.
